# German version, inter- and intrarater reliability and internal consistency of the “Agitated Behavior Scale” (ABS-G) in patients with moderate to severe traumatic brain injury

**DOI:** 10.1186/s12955-016-0511-x

**Published:** 2016-07-19

**Authors:** Stephanie Hellweg, Corina Schuster-Amft

**Affiliations:** Department of Neurological Rehabilitation, Rehaklinik Bellikon, Bellikon, 5454 Switzerland; Institute of Physiotherapy, School of Health Professions, Zurich University of Applied Sciences, Winterthur, 8400 Switzerland; Research Department, Reha Rheinfelden, Salinenstrasse 98, Rheinfelden, 4310 Switzerland; Institute for Rehabilitation and Performance Technology, Bern University of Applied Sciences, Pestalozzistrasse 20, Burgdorf, 3400 Switzerland

**Keywords:** Traumatic brain injury, Agitation, Agitated behavior scale, Interrater reliability, Intrarater reliability, Internal consistency

## Abstract

**Background:**

Agitation is frequently observed during early recovery after traumatic brain injury (TBI). Agitated behaviour often interferes with a goal-orientated rehabilitation and can be a substantial hindrance to therapy. Despite the relatively high occurance of agitation in TBI population there is no objective assessement in German (G) available. An existing scale with excellent psychometric properties is the “Agitated Behavior Scale (ABS)” developed by Corrigan in 1989. The aim of the study was to translate the Agitated Behavior Scale (ABS) into German (ABS-G) and investigate the inter- and intrarater reliability and internal consistency in patients with moderate to severe TBI.

**Methods:**

A formal nine-step translation and cross-cultural adaptation procedure (TCCA) was applied. Subsequently a prospective observational patient study was conducted. To examine the interrater reliability and internal consistency, two therapists rated 20 patients independently after a therapy session. This procedure was repeated twice on a weekly basis. The intrarater reliability was assessed through video recordings from three patients. Nine raters scored the demonstrated behaviour on the videotape with the ABS-G independently twice within one month. The inter- and intrarater reliability were evaluated with the Spearman rank correlation coefficient and the quadratic weighted kappa. The internal consistency was tested with Cronbach’s alpha.

**Results:**

Behaviour of 20 patients (18 males; mean age 41 ± 20.7; mean Functional Independence Measure (FIM) cognitive score on admission 7.1 ± 4.04; mean ABS-G score at first observation 17.3 ± 2.83) was assessed threefold. Interrater reliability yielded a correlation coefficient for ABS-G total score of all 60 paired observations of *r*_*s*_ 0.845 and a weighted Kappa of 0.738. Intrarater reliability for ABS-G total score ranged between *r*_*s*_ 0.719 and 0.953 and showed a weighted Kappa between 0.871 and 0.953. Cronbach’s alpha indicated moderate internal consistency with 0.661.

**Conclusion:**

This study demonstrates that the ABS-G is a reliable instrument for evaluating agitation in patients with moderate to severe TBI. Hereby it would be possible to monitor agitation objectively and optimise the management of agitated patients according to international recommendations.

## Background

Agitation is frequently observed during the early stage of recovery after moderate to severe traumatic brain injury (TBI) [1, 2]. Agitation is characterised by behavioural disturbances such as disinhibition expressed for example as restlessness, pulling at tubes etc., impulsivity, emotional lability, verbal or physical aggression, perseveration and confusion [3–5]. Although agitated behaviour is usually brief in duration (1–14 days) and disappears prior to resolution of posttraumatic amnesia [2], it can also be a long-lasting problem [6]. Recent evidence proposes a prevalence of this behaviour in TBI patients between 35 and 45 % [7, 8].

Agitation plays an important role in the rehabilitation process and interferes with everyday functioning, treatment compliance and hinders a goal-directed rehabilitation [9, 10]. The burden for family, relatives, and the interdisciplinary treating team is substantial and commonly leads to distress [3, 11]. In addition, agitated behaviour is associated with a prolonged length of stay, a reduced functional independence at discharge [10], and higher costs if constant observation is necessary [12]. Despite the relatively high frequency and the serious impact of this phenomenon the authors are not aware of an assessment in German (G) to evaluate agitation in a standardised way.

One well-investigated scale to assess agitation is the English “Agitated Behavior Scale” (ABS) [13]. Studies have shown sound psychometric properties of the English version with excellent interrater reliability (*r* = 0.92) and fair internal consistency (Cronbach’s alpha between 0.801 and 0.921) [13, 14]. Content, concurrent and construct validity likewise have been tested. The conducted factor analysis revealed a general construct with three underlying factors: aggression, disinhibition, and lability [13–16]. The use of the ABS to evaluate agitation following TBI was recently recommended by an international expert panel [5]. The aims of the present study were 1) developing a German version of the ABS (ABS-G) using the proposed translation and cross-cultural adaptation procedure (TCCA) and 2) examine the inter- and intrarater reliability and internal consistency of the ABS-G in patients with moderate to severe TBI.

## Methods

### Design

#### The translation and cross-cultural adaptation procedure

A formal nine-step TCCA was defined and implemented for the ABS-G (Table 1; steps 1–9) according to existing recommendations and guidelines such as the ISPOR principles of good practice [17–19]. All articles recommended a systematic multi-step approach including more than one forward translation to detect errors and divergent interpretations of ambiguous items in the original. Since the ABS is used interdisciplinary three forward and two backward translations were obtained, done by a physician, an occupational therapist (OT), a movement scientist and a professional translator. All were native speakers of the target language. Compared to Schuster’s procedure [18], the TCCA was slightly modified to reveal and deal with any translation discrepancies that arise from different professional backgrounds of the translators. Thus an expert committee review with 4 out of 5 translators was carried out to ensure conceptual equivalence between the source and target language versions. Table 1 summarized all individual steps of TCCA.

**Table 1:** Nine-step procedure for translation and cross-cultural adaptation of the Agitated Behavior Scale

Step	Aim	Required personnel
1	To obtain permission to use instrument and to invite instrument developer to be involved.	First author
2	To receive three independent forward translations and make necessary cross-cultural adaptations of the item content, scoring system and scoring instructions into the target language.	The two informed^a^ translators and the naive^b^ translator were native speakers of the target language
3	To synthesize the forward translation into a single forward translation and to resolve any discrepancies with translators’ reports. To review layout, grammar and typography.	The synthesis was done by the first author
4	To back translate the reconciled translation into the source language by two English native speaker.	The two back translators, one informed^a^and one naive^b^, were bilingual and unaware of the original ABS
5	To synthesize the backward translation into a single backward translation and to detect conceptual errors, inconsistencies, unclear wording, equivalence (semantic and idiomatic) of the translation. To review layout, grammar and typography.	The first author did the synthesis and review.
	To review the back translation by the original author.	Original author
6	Expert committee review: To review all reports in a committee and to reach consensus on discrepancies. To achieve semantic and idiomatic equivalence of the forward translation. To produce pre-final version: to review layout, grammar and typography (adapt and re-check).	*Expert committee: Four of five translators were present at the meeting as well as the first author and the two pre-testing professionals*
7	To pre-test the translated version with two patients including the comprehension of item content, scoring system and scoring instructions and to detect difficulties or discrepancies within the translated version. To finalize the translation and to proof the finalized translation	Two professionals have tested the pre-test version with patients and have written a short report. The first author has integrated their feedback in the finalized version. Proof-reading was done by a professional translator
8	To report the process of translation.	First author
9	To evaluate the inter-, intrarater reliability and internal consistency.	Patients, therapists, first author

^a^ an informed translator has expertise of the underlying concepts being tested by the assessment and has a medical or clinical background

^b^ a naive translator has no inside in the underlying concept and has no clinical background. Favourably, the naive translator has expertise in translation

#### Implementation of the TCCA procedure

##### Step 1

Preparation stage: the first author developed a detailed translation procedure and defined the translator requirements. The permission of the original instrument developer was obtained and translators were recruited.

##### Step 2

The English ABS was forward translated by three native German speakers with excellent English language skills. Two out of three translators were aware of the study objectives. As suggested by Beaton et al., one of the translators was a naive translator, who was not aware nor informed of the concepts being quantified and had no medical or clinical background.

##### Step 3

The forward translations were synthesized and checked for correct grammar, typography and layout by the first author and re-checked by the professional translator, who was the naive translator of step 2.

##### Step 4

The forward translation was back translated by two bilingual translators. One was a research assistant with experience in the TCAA procedure and one was a professional translator.

##### Step 5

The backward translations were synthesized and checked for correct grammar, typography and layout by the first author and rechecked by a one of the forward translators. All documents were send to the original author for review.

##### Step 6

Four out of five translators and the first author participated in an expert committee review. In addition, two professionals took part, who pre-tested the ABS-G subsequent to this step. The focus of the expert review was the discussion of divergent translations and interpretations of ambiguous items. Consensus was achieved by discussion. In case of disagreement majority decisions were obtained.

##### Step 7

The German version was pre-tested by two therapists (PT, OT) with three patients before starting the patient study to determine practical feasibility and applicability.

##### Step 8

The final translation report was written by the first author to explain the reasons for translation/wording choices made throughout the translation process.

##### Step 9

A prospective observational patient study (Table 1; step 9) was carried out to assess inter- and intrarater reliability and the internal consistency of the German version of the ABS (ABS-G). The procedure adhered to the guidelines for reporting reliability and agreement studies (GRRAS) and the consensus-based standards for the selection of health measurement instruments (COSMIN) guidelines [20–22].

#### Ethics

The study was conducted in accordance with the International Conference on Harmonisation-Good Clinical Practise (ICH-GCP) guidelines and the Helsinki Declaration. The ethics committee of Northwest and Central Switzerland approved the protocol (EKNZ 2014-120). Due to study design no trial registration was demanded. Written informed consent was obtained for using patient data from the legal representative of the patient or from the patients if judicious.

#### Outcome measures and material

Similar to the original version the translated ABS-G (Appendix 1) consists of 14 items characterising different types of behaviour. The items are rated on an ordinal scale from 1 (absent) to 4 (present to an extreme degree). A total score of ≤21 points is rated as normal behaviour, 22-28 points as mild agitation, 29–35 points as moderate agitation and a score ≥36 points as severe agitation [23]. The scale comprises three subscales: aggression, disinhibition and lability [16] with the total score as the best overall measure of agitation [23].

Furthermore, data were collected for patients’ age, gender, diagnosis, type of accident, time from accident to admission in the neurological rehabilitation department and the functional independence measure (FIM) total and cognitive score at admission. FIM cognitive score was chosen since it predicts more severe agitation [24].

#### Participants and study settings

Patients with moderate to severe TBI as defined by a Glasgow Coma Scale (GCS) score at the time point of screening for eligibility, who had been directly transferred from an intensive or intermediate care unit, were consecutively screened for eligibility when admitted to the 65-bed neurological rehabilitation department between July and December 2014. The inclusion criteria were as follows:

- patients with moderate to severe TBI as defined by a GCS score between 4 and 12 or with GCS of 13–14 and severe focal neurological deficits and/or severe agitation at the time point of screening for eligibility,
- injury occurred within 12 months before admission, and
- ≥16 years old.

Patients were excluded if they presented a non-traumatic brain injury, (e.g. tumour, stroke) or a GCS score of three.

#### Raters

Data collection was performed by six physical therapists (PT), five occupational therapists (OT) and one nurse specialised in dysphagia (DT) with at least 5.5 years professional experience and a minimum of 3.5 years experience in TBI rehabilitation. The first author trained all raters for at least 60 min, including a presentation of the ABS-G and rating examples constructed by the ABS-original authors.

#### Data collection procedure

##### Interrater reliability agreement/internal consistency

Two raters simultaneously conducted the scoring of the ABS-G, subsequent to a routine joint therapy session. The ABS-G ratings were filled in independently and no communication was allowed. In total, 20 patients were assessed at three points in time, resulting in 60-paired ratings. The time interval between ratings was approximately one week. The pairing of therapists conducting the therapy session was freely selected by the therapy planning team (convenient composition), therefore the constellation of the pair of raters was arbitrary. A sample size of 60-paired ratings was chosen in concordance with guideline recommendations [21, 22].

##### Intrarater reliability

Three patients with different levels of agitation following TBI were video recorded for 10 min during a treatment session. The duration of 10 min observational interval was chosen referring to Bogner et al. [14]. Nine raters (five PTs, three OTs, one DT) independently scored the videotaped behaviour with the ABS-G on two occasions. Between the two ratings of each rater, a minimum period of one month helped to minimize possible memory effects. In total, 27-paired ratings were obtained, resulting in 54 ratings.

#### Statistical analysis

The psychometric properties of the ABS-G were evaluated for (1) interrater reliability, (2) intrarater reliability and (3) internal consistency. For sample characteristics, descriptive statistics were used. Patient characteristics were described using means and SD for continuous variables and frequency and percentages for categorical variables.

To access (1) interrater and (2) intrarater reliability Spearman rank correlation coefficients (*r*_*s*_*)* were calculated to establish the agreement between the two raters (1) respectively between the two ratings (2) of the ABS-G total score. (1) Interrater and (2) intrarater reliability of the ordinal scores on item level were determined by weighted Kappa (*wk*) [25], using a quadratic weighting scheme. For comparison reason with reliability studies of other authors [8, 14] Pearson product moment correlation coefficient (*r*) as well as intraclass correlation coefficients ICC (1,1) using a one-way random model with absolute agreement were calculated. (3) Cronbach’s alpha (α) was calculated to describe the internal consistency of the ABS-G. Statistically significant difference was set at *p* < 0.05 for all analyses. All statistical analyses were performed with the statistical package for social sciences, SPSS (Version 22.0, IBM, Armonk NY, USA).

## Results

### TCCA procedure

The TCCA procedure was realised as defined. Regarding the used vocabulary there was a difference between the three forward translations. Particularly the expert committee review was helpful to discuss linguistic discrepancies due to the different professional backgrounds of the translators (physician, OT, and professional translator). In addition, the review helped to reach consensus regarding the wording, for instance the word “extreme” in the scoring system. It is uncommon in German speaking countries to use “extreme” in the context of a scoring systems, therefore the committee agreed to replace the term “extreme” with “strong”.

### Participants

During the recruitment phase 43 patients were screened for eligibility. Totally, 20 patients (18 male) met the inclusion criteria. The mean reason for exclusion was a high GCS score (over 12 respectively 14) at the time point of screening for eligibility. The Mean age of the subjects was 41 ± 20.7 years (range 16–83) and 17 out of 20 patients experienced a severe TBI. The mean total FIM score on admission was 31.2 ± 25.4 (range 18–107), whereby 13 patients showed a total FIM admission score under 20. The mean cognitive FIM score on admission was 7.1 ± 4.04 (range 5–22). Time since injury ranged from 10 to 50 days with a mean of 24.2 days. The mean ABS-G score at the first observation was 17.3 ± 2.8. Mild agitation was observed in 5–15 % of patients over the three points in time (Tables 2 and 3). There were no missing values.

**Table 2:** Patient Characteristics (*N* = 20)

Characteristic	Number	Percentage
Gender		
male	18	90 %
females	2	10 %
Age (y)		
16–39	11	55 %
50–60	5	25 %
>70	3	15 %
Diagnosis (classified using the GCS score)		
severe TBI	17	85 %
moderate TBI	1	5 %
not defined TBI (upon hospital admission)	2	10 %
Injury cause		
falls	7	35 %
transport	11	55 %
others (gunshot, work)	2	10 %
FIM total score admission		
<20	13	65 %
20–50	3	15 %
51–80	3	15 %
>80	1	5 %
FIM cognitive score admission		
<10	16	80 %
11–20	3	15 %
>21	1	5 %

**Table 3:** Patients’ ABS-G ratings at time point 1

Item	1	2	3	4	5	6	7	8	9	10	11	12	13	14	ABS-G	FIM-C	FIM-T
p1	2	2	1	1	1	1	3	2	2	1	1	1	1	1	20	5	18
p2	1	1	2	1	1	1	1	1	1	1	1	1	1	1	15	5	18
p3	1	1	1	1	1	1	1	1	1	1	1	1	1	1	14	5	18
p4	3	1	1	1	1	1	1	1	1	4	3	1	3	1	23	5	18
p5	2	1	1	1	1	1	1	1	1	1	1	1	1	1	15	7	23
p6	4	1	3	4	1	1	4	1	4	1	1	1	1	1	28	5	18
p7	1	1	1	1	1	1	1	1	1	2	1	1	1	1	15	12	79
p8	1	1	1	1	1	1	1	1	1	1	1	1	1	1	14	5	18
p9	3	1	1	1	1	1	1	1	1	1	4	1	1	1	19	6	54
p10	3	1	1	1	1	1	1	1	1	2	3	1	1	1	19	5	18
p11	1	1	1	1	1	1	1	1	1	1	1	1	1	1	14	8	30
p12	1	1	1	1	1	1	1	1	1	1	1	1	1	1	14	5	18
p13	2	1	1	1	1	1	1	1	1	1	3	1	1	1	17	12	69
p14	1	1	1	1	1	1	1	1	1	1	1	1	1	1	14	6	19
p15	1	1	1	1	1	1	1	1	1	1	1	1	1	1	14	5	18
p16	1	4	1	1	2	1	2	1	1	1	1	2	1	1	20	5	18
p17	1	2	1	1	1	1	1	1	1	1	3	1	1	1	17	22	107
p18	1	2	1	1	1	1	1	1	1	1	1	1	1	1	15	8	27
p19	4	1	1	1	1	1	1	1	4	1	1	1	1	1	20	5	18
p20	3	1	1	1	1	1	1	1	1	2	1	1	1	1	17	5	18

Legend: *p* patient, *ABS-G* total score German version Agitated Behavior Scale, *FIM-C* Functional Independence Measure, Cognitive Subscale, *FIM-T* Functional Independence Measure, Total Score

1. ***Interrater reliability***
  Interrater reliability ratings (Table 4) yielded a correlation coefficient for ABS-G total score of all 60-paired observations of *r*_*s*_ 0.845, *p* < 0.001. On item level, *qwk* demonstrated the substantial interrater reliability of 0.738.

**Table 4:** Interrater reliability

	Total score all TP	Total score TP1	Total score TP2	Total score TP3	Item-score all TP	Item-score TP1	Item-score TP2	Item-score TP3
*r*_*s*_	.845	.823	.936	.802	.691	.636	.772	.662
(*r*)	.899	.758	.897	.857	.738	.745	.791	.646
(ICC_1.1_)	.899	.925	.897	.860	.739	.746	.791	.644
*qwk*	-	-	-	-	.738	.744	.790	.643

Legend: *r*_*s*_ *=* Spearman rank correlation coefficient; *r* = Pearson product moment correlation coefficient; ICC_1.1_ = Intraclass correlation coefficients; *qwk* = quadratic weighted kappa; TP = Time Point
2. ***Intrarater reliability***
  Intrarater reliability ratings for the ABS-G total score of two ratings *r*_*s*_ ranged for all raters between 0.719 and 0.953, *p* < 0.001. For details see Table 5. On item level, *qwk* illustrated almost perfect intrarater reliability agreement with values ranging between 0.871 and 0.963.

**Table 5:** Intrarater reliability

	Rater 1	Rater 2	Rater 3	Rater 4	Rater 5	Rater 6	Rater 7	Rater 8	Rater 9
*r*_*s*_	.912	.953	.883	.814	.914	.879	.886	.783	.719
*qwk*	.963	.963	.880	.871	.919	.955	.888	.898	.912

Legend: *r*_*s*_ *=* Spearman rank correlation coefficient; *qwk* = quadratic weighted kappa
3. ***Internal consistency***
  Cronbach’s alpha of the total score of ABS-G indicated moderate internal consistency with α = 0.661. In particular, the correlation between item 8, respectively item 11 and the sum of the rest of the items show low item selectivity. If item 11 (rapid talking) would be deleted, α would rise to 0.689 (Table 6).

**Table 6:** Cronbach’s alpha

		Corrected^a^ item scale correlation	Cronbach’s alpha if item deleted
Item 1	Distractibility	.534	.590
Item 2	Impulsivity	.456	.610
Item 3	Resistance to care	.410	.632
Item 4	Violence	.486	.615
Item 5	Anger	.231	.655
Item 6	Self-stimulation	.242	.652
Item 7	Tube pulling	.300	.643
Item 8	Wandering	.060	.664
Item 9	Restlessness	.477	.608
Item 10	Repetitiveness	.278	.647
Item 11	Rapid talking	.064	.689
Item 12	Sudden mood change	.197	.656
Item 13	Crying/laughing	.159	.659
Item 14	Self-abusive	.116	.663

Legend: ^a^Correlation between each item and the scale score excluding that item
  Similar to the original version Cronbach’s alpha of the subscales aggression, disinhibition and lability is lower with α = 0.543, α = 0.637 respectively α = 0.259, indicating that the total score remains the best overall measure of agitation [23].

## Discussion

The aim of this study was to provide an official German version of the ABS as an objective and reliable tool for assessing agitation in traumatic brain-injured patients recovering from coma. For this purpose a formal nine-step TCCA procedure was applied. Different from the TCCA procedure for objectively-assessed outcome measures (OAO) [18], Beaten’s recommendation [17] regarding the requirements for translators was followed since the ABS is used interdisciplinary by diverse professionals (not profession-specific).

The present study’s results suggest a high correlation between raters of the total score with *r*_*s*_ *=* 0.845 and a substantial interrater reliability [26] on item level with a *qwk =* 0.738. In comparison to the English version [14], values are slightly lower (*r* = 0.920). This might be explained by the smaller sample size (20 patients compared to 45 subjects in [14]). Additionally, the studies’ settings differed: Bogner et al. [14] asked research assistants to observe activities on the nursing unit or in therapeutic sessions, enabling them to focus on the patients’ behaviour only. In the present study, therapists treated the patient and had to fill in the ABS-G afterwards. Furthermore, a pair of mixed therapists (OT, PT, DT) evaluated the patients’ demonstrated behaviour. Therefore, the different professional backgrounds of therapists might explain the slightly lower correlation (*r* = 0.899 in the present study versus *r* = 0.920 [14]) of interrater reliability. These findings are in line with previous findings regarding the ABS: Monodisciplinary ratings yielded a correlation coefficient of 0.920, multidisciplinary ratings showed a correlation range between 0.364 and 0.604 [14]. However, since the management of agitated patients usually is organised interdisciplinary, a high correlation between different therapists is pertinent to everyday clinical practise.

The inclusion and exclusion criteria of patients have to be critically discussed. In order to include all patients, who pass through a confusional state with possible agitation we decided to use broad inclusion criteria, even though patients with a GCS score under 8 might show no agitation. On the other hand the termination of sedation is a typical procedure during early rehabilitation. When patients subsequently regain consciousness the majority experiences a period of confusion, not seldom accompanied by agitation as they regain awareness [5].

This is the first study that provided an official German version of the ABS and examined its intrarater reliability. Our results suggest a high correlation between repeated measurements of the total score with *r*_*s*_ between 0.719 and 0.912 and an almost perfect *qwk* between 0.871 and 0.963 [26].

Studies have shown the ABS to be highly internally consistent (Cronbach’s alpha between 0.801 and 0.921) [13, 15]. Our result with α = 0.661 implies only moderate internal consistency of the ABS-G. One reason for this difference might be the observational setting. Our data collection involved a therapeutic session inhibiting certain behaviours such as wandering from treatment areas (item 8). Additionally, the present study involved patients with a low FIM score (FIM < 20 in 13 patients). Hence a number of patients were unable to display certain behaviours such as wandering and rapid talking (item11) due to their inability to walk or communicate. Based on the low FIM score the variance of some items between patients was relatively low, thus lowering Cronbach’s alpha. Furthermore the construct of agitation as illustrated in the scale is very heterogeneous, lowering the inter-item correlation as you can see in Table 6. According to Streiner [27] it is recommended to sacrifice internal consistency to content validity if the scale measures a heterogeneous construct as behavior. Hence Cronbach’s alpha of 0.661 is acceptable.

This study had some limitations. Sample size was small, which prohibited the analysis of construct validity through factor analysis. Furthermore responsiveness had not been established.

## Conclusion

The present study confirms the inter- and intrarater reliability as well as the internal consistency of the ABS-G. On this basis monitoring of agitation during the rehabilitation process will become possible as international recommendations suggest [5]. Particularly the impact of new strategies or therapies on the patient’s level of agitation can be critically evaluated. Further studies should examine the validity of the ABS-G and the transferability particularly in the nursing context and with other patient populations such as dementia.

## Abbreviations

ABS, Agitated Behavior Scale; ABS-G, Agitated Behavior Scale - German; DT, Dysphagia Therapist; EKNZ, Ethics Committee of Northwest and Central Switzerland; FIM, Functional Independence Measure; G, German; GCS, Glasgow Coma Scale; ICH-GCP, International Conference on Harmonisation-Good Clinical Practise; ISPOR, International Society of Pharmacoeconomics and Outcomes Research; OT, Occupational Therapist; PT, Physical Therapists; *qwk*, quadratic weighted Kappa; *r*, Pearson Product Moment Correlation Coefficient; *r*_*s*_, Spearman Rank Correlation Coefficient; SPSS, Statistical Package for Social Sciences; TBI, Traumatic Brain Injury; TCCA, Translation and Cross-Cultural Adaptation; α, Cronbach’s alpha.

## Appendix 1

Agitated Behavior Scale – German (ABS-G) (Skala zur Erfassung psychomotorischer Unruhe)

**Table:**

Patient:	Datum:
Beobachtungskontext:	Uhrzeit: von__Uhr bis__ Uhr
Beurteiler:	

Geben Sie am Ende des Beobachtungszeitraums an, ob das beschriebene Verhalten im entsprechenden Punkt vorhanden war und, falls ja, in welchem Ausmass: leicht, mässig oder stark. Für die Bewertung sind folgende Kriterien und numerischen Abstufungen anzuwenden.

**Table:**

1	**nicht vorhanden**: das Verhalten kommt nicht vor.
2	**in leichter Ausprägung vorhanden**: das Verhalten kann beobachtet werden, aber es verhindert nicht anderes, situationsbedingt angemessenes Verhalten (entweder zeigt die Person spontan eine Verhaltensanpassung, oder das Fortbestehen des agitierten Verhaltens verunmöglicht ein angemessenes Verhalten nicht).
3	**in mässiger Ausprägung vorhanden**: die Person benötigt Unterstützung um vom agitierten Verhalten zu einem angemessenen Verhalten zu finden, aber profitiert von entsprechenden Hinweisen.
4	**in starker Ausprägung vorhanden**: die Person kann aufgrund des agitierten Verhaltens nicht zu einem angemessenes Verhalten finden, selbst wenn von extern ein Hinweis oder Anstoss zur Verhaltensänderung gegeben wird.

*Bitte keine Beurteilung unausgefüllt lassen:*

**Table:**

**Punktzahl von 1–4**		**Verhalten**
	1	Kurze Aufmerksamkeitsspanne, leichte Ablenkbarkeit, Unfähigkeit, sich zu konzentrieren
	2	Impulsiv, ungeduldig, geringe Schmerz- oder Frustrationstoleranz
	3	Unkooperativ, verweigert sich pflegerischen Massnahmen, fordernd
	4	Gewalttätig und/oder droht mit Gewalt gegen Personen oder Gegenstände
	5	Explosiv und/oder unvorhersehbare Wutausbrüche
	6	Schaukeln, Reiben, Stöhnen oder andere selbststimulierende Verhaltensweisen
	7	Ziehen an Schläuchen, Schutzeinrichtungen (z. B. Fixationsgurten) etc.
	8	Verlassen von Behandlungsräumen
	9	Unruhe, Hin- und Hergehen, übermässiges Bewegen
	10	Repetitives Verhalten, motorisch und/oder verbal
	11	Schnelles, lautes oder übermässiges Sprechen
	12	Plötzliche Stimmungswechsel
	13	Leicht auslösbares oder übermässiges Weinen und/oder Lachen
	14	Selbstverletzende Handlungen oder Selbstbeschimpfungen
		**Totale Punktzahl**
